# Trypanosoma brucei L11 Is Essential to Ribosome Biogenesis and Interacts with the Kinetoplastid-Specific Proteins P34 and P37

**DOI:** 10.1128/mSphere.00475-19

**Published:** 2019-08-21

**Authors:** Daniel Jaremko, Martin Ciganda, Linda Christen, Noreen Williams

**Affiliations:** a Department of Microbiology and Immunology, University at Buffalo, Buffalo, New York, USA; Carnegie Mellon University

**Keywords:** 5S rRNA, L11, protein-RNA interactions, protein-protein interactions, ribosomal proteins, ribosome biogenesis, trypanosomes

## Abstract

Eukaryotic ribosome biogenesis is an essential cellular process involving tightly coordinated assembly of multiple rRNA and protein components. Much of our understanding of this pathway has come from studies performed with yeast model systems. These studies have identified critical checkpoints in the maturation of the large ribosomal subunit (LSU/60S), one of which is the proper formation and incorporation of the 5S ribonucleoprotein complex (5S RNP). Research on the 5S RNP has identified a complex containing the four proteins L5, L11, Rpf2, and Rrs1 as well as 5S rRNA. Our laboratory has studied the 5S RNP in Trypanosoma brucei, a eukaryotic parasite, and identified the proteins P34 and P37 as essential, parasite-specific members of this complex. We have additionally identified homologues of L5, Rpf2, Rrs1, and 5S rRNA in T. brucei and characterized their roles in this essential process. In this study, we examined the T. brucei homologue of ribosomal protein L11 as a member of the 5S RNP. We showed that TbL11 is essential and that it is important for proper ribosome subunit formation and 60S rRNA processing. Additionally, we identified TbL11 interactions with TbL5 and TbRpf2, as well as novel interactions with the kinetoplast-specific proteins P34 and P37. These findings expand our understanding of a crucial process outside the context of model yeast organisms and highlight differences in an otherwise highly conserved process that could be used to develop future treatments against T. brucei.

**IMPORTANCE** The human-pathogenic, eukaryotic parasite Trypanosoma brucei causes human and animal African trypanosomiases. Treatments for T. brucei suffer from numerous hurdles, including adverse side effects and developing resistance. Ribosome biogenesis is one critical process for T. brucei survival that could be targeted for new drug development. A critical checkpoint in ribosome biogenesis is formation of the 5S RNP, which we have shown involves the trypanosome-specific proteins P34 and P37 as well as homologues of Rpf2, Rrs1, and L5. We have identified parasite-specific characteristics of these proteins and involvement in key parts of ribosome biogenesis, making them candidates for future drug development. In this work, we characterized the T. brucei homologue of ribosomal protein L11. We show that it is essential for parasite survival and is involved in ribosome biogenesis and rRNA processing. Furthermore, we identified novel interactions with P34 and P37, characteristics that make this protein a potential target for novel chemotherapeutics.

## Abstract (precis)

The human-pathogenic, eukaryotic parasite Trypanosoma brucei causes human and animal African trypanosomiases. Treatments for T. brucei suffer from numerous hurdles, including adverse side effects and developing resistance. Ribosome biogenesis is one critical process for T. brucei survival that could be targeted for new drug development. A critical checkpoint in ribosome biogenesis is formation of the 5S RNP, which we have shown involves the trypanosome-specific proteins P34 and P37 as well as homologues of Rpf2, Rrs1, and L5. We have identified parasite-specific characteristics of these proteins and involvement in key parts of ribosome biogenesis, making them candidates for future drug development. In this work, we characterized the T. brucei homologue of ribosomal protein L11. We show that it is essential for parasite survival and is involved in ribosome biogenesis and rRNA processing. Furthermore, we identified novel interactions with P34 and P37, characteristics that make this protein a potential target for novel chemotherapeutics.

## INTRODUCTION

Trypanosoma brucei is a single-celled, eukaryotic parasite responsible for the diseases human African trypanosomiasis (HAT) and animal African trypanosomiasis (AAT). These two diseases pose serious health and economic burdens in sub-Saharan African countries where the vector of T. brucei, the tsetse fly, is endemic (1). While there are some therapeutic treatments available for HAT and AAT, these treatments have significant problems, including cost, adverse side effects, and evidence of developing resistance (2–4). As a result, there is a continuing need for research to identify potential new drug targets to combat these diseases. One approach is to focus on pathways that both are essential and contain parasite-specific components, such as ribosome biogenesis.

Eukaryotic ribosome biogenesis has largely been studied in the yeast Saccharomyces cerevisiae, in which extensive and ongoing research has revealed it to be a complex process. This process involves four rRNAs, over 90 ribosomal proteins, and around 200 assembly factors, all acting in a concerted and stepwise fashion (5). Proper assembly of these components is required to generate functional ribosomes, and its disruption halts ribosome biogenesis and results in degradation of the aberrant intermediates.

To ensure proper ribosome maturation, a number of critical steps need to occur at appropriate points in assembly. One of these is the formation and incorporation of the 5S ribonucleoprotein complex (5S RNP), a protein-RNA neighborhood that is critical to early stages of 60S formation (6). In S. cerevisiae, the nucleolar ribosome-associating 5S RNP is comprised of the 5S rRNA, the ribosomal proteins L5 and L11, and the assembly factors Rrs1 and Rpf2. Failure to incorporate any of these components into the 5S RNP interrupts assembly of the 60S subunit, acting as an important checkpoint in ribosome biogenesis (6).

Work from our laboratory has shown that the trypanosome-specific proteins P34 and P37 are essential in T. brucei (7). Furthermore, loss of P34 and P37 results in a disruption of ribosome biogenesis, with an increase of 60S subunits and consequent decrease in 80S subunits, supporting their role in 60S maturation (7). A decrease in P34 and P37 also leads to a decrease in 5S rRNA abundance (7). Further evidence showed that P34 and P37 directly bind to 5S rRNA *in vivo* (8) and *in vitro* (9). In addition, P34 directly interacts *in vitro* and *in vivo* with the T. brucei homologues of L5 (10), Rpf2 (11), and Rrs1 (12), further strengthening its position as a trypanosome-specific member of the 5S RNP. This makes the assembly of the 5S RNP a promising target for study in T. brucei due to both its critical nature and the presence of trypanosome-specific proteins P34 and P37.

While the T. brucei 5S RNP, and P34 and P37 in particular, is a promising target for drug development, the potential role of the T. brucei L11 homologue (TbL11) in the 5S RNP has not yet been examined. Ribosomal protein L11 has been largely studied in S. cerevisiae, in which research has shown it to be an essential protein that is important to 60S subunit (13) and 25S rRNA maturation (14). In addition, L11 has important functional roles in probing the occupancy of the 60S P-site (15) and transmitting information between the 40S and 60S subunits via bridging connections with 40S components (16). Furthermore, in S. cerevisiae L11 directly interacts with Rpf2 (17), L5 (6, 18), and Rrs1 (19), confirming it as a member of the nucleolar ribosome-associating 5S RNP. Recent work has focused on the role of L11 in modulating the p53-MDM2 pathway in human cells and its relevance to the pathogenesis of certain ribosomopathies and cancers (20, 21). This suggests a broader importance of L11 that might not be limited to ribosome biogenesis in S. cerevisiae but could also be relevant to other organisms and other processes.

With this in mind, our laboratory was interested in examining the potential role that TbL11 (TriTryp accession numbers Tb927.9.7590 and Tb927.9.7620, identically coded proteins) might play in 5S RNP formation in the unique context of a complex containing the trypanosome-specific proteins P34 and P37. We first developed RNA interference (RNAi) cell lines for TbL11 to determine the impact of the loss of TbL11 on cell survival and morphology. We then determined whether the expression of other 5S RNP members (P34 and P37, TbL5, and TbRpf2) was altered at the level of protein abundance in the absence of TbL11. Next, we used the abundance of ribosomal subunits as well as shifts in rRNA processing to determine where and when the targeted proteins might act in T. brucei ribosome biogenesis. Finally, we used a number of *in vivo* and *in vitro* assays to determine which members of the 5S RNP interact with TbL11.

Taken together, these studies have shed new light on a critical portion of ribosome biogenesis in the eukaryotic parasite T. brucei. By examining how TbL11 fits into this process in the context of the trypanosome-specific components, we have expanded the map of nucleolar ribosome-associating 5S RNP formation in T. brucei to allow future development of trypanocidal drugs that act upon this critical part of ribosome biogenesis.

## RESULTS

### TbL11 associates with 5S rRNA, L5, and P34 and P37 *in vivo*.

We began by identifying binding partners of 5S rRNA *in vivo*. We incubated biotinylated 5S rRNA with a cell lysate to select for associating proteins that were then analyzed using mass spectrometry. The results (see Data Set S1 in the supplemental material, highlighted proteins) included P34 and P37 and TbL5, which we have previously identified as members of the 5S RNP in T. brucei (10) and as strong binding partners of 5S rRNA (9, 22). Interestingly, we also observed the presence of ribosomal protein TbL11, which has not previously been characterized in T. brucei. Furthermore, previous studies from our laboratory involving the *in vivo* association of proteins with tagged TbL5, tagged P34, and tagged P37 also identified TbL11 as a binding partner of these three members of the T. brucei 5S RNP (11). Taken together, our affinity capture assays indicated that TbL11 might be an integral part of the T. brucei 5S RNP and might interact with trypanosome-specific proteins P34 and P37.

10.1128/mSphere.00475-19.1 DATA SET S1 5S rRNA affinity purification data. Tables show the filtered results for the 5S rRNA affinity pulldown assay, categorized by 60S ribosomal proteins, 40S ribosomal proteins, hypothetical proteins, and the remaining proteins. Proteins relevant to this work are highlighted. Download Data Set S1, XLSX file, 0.05 MB. Copyright © 2019 Jaremko et al. 2019 Jaremko et al. This content is distributed under the terms of the Creative Commons Attribution 4.0 International license.

### TbL11 is an essential protein in T. brucei.

**FIG 1:**
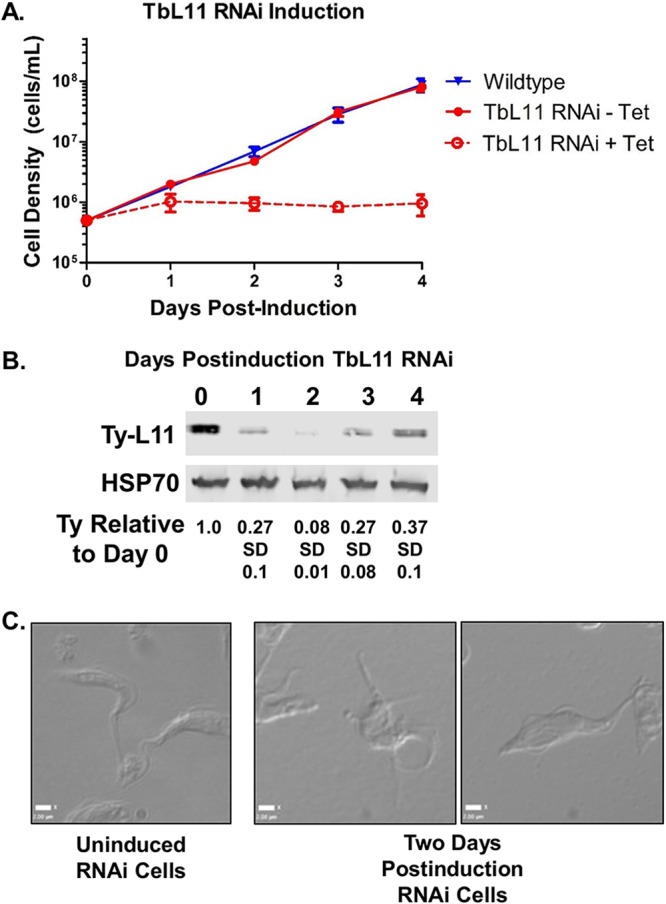
TbL11 is an essential protein for T. brucei survival. (A) Growth curves were calculated comparing wild-type cells versus uninduced (-Tet [tetracycline]) and induced (+Tet) TbL11 RNA interference cells. (B) Western blot analysis was performed on RNAi cell extracts collected at days 0 to 4 after induction of TbL11 RNAi using HSP70 as a loading control. (C) DIC microscopy was used to capture images of cells with or without TbL11 RNA interference induction. Analyses were performed on three biological replicates, with representative samples shown.

We next developed both Ty-tagged and untagged RNA interference (RNAi) cell lines targeted against the TbL11 transcripts. Induction of TbL11 RNAi in both cell lines resulted in a noticeable growth defect at 2 days postinduction that continued throughout 4 days postinduction (Fig. 1A and Fig. S1). Furthermore, we were able to show via Western blotting against Ty-tagged L11 (Fig. 1B) that knockdown significantly reduced levels of TbL11 protein {to 0.11 (standard deviation [SD], 0.03) by 2 days postinduction relative to uninduced cells}. Cell lines containing either tagged or untagged TbL11 showed escape from the RNAi, with TbL11 protein (detected as tagged TbL11) increasing by days 3 and 4 and cell growth increasing by days 7 and 8 (Fig. S1). While differential interference contrast (DIC) images of cells taken before induction of RNAi showed a normal, wild-type morphology, the cells became bloated, multiflagellated, and misshapen by 2 days postinduction (Fig. 1C). Taken together, these results indicate that TbL11 is essential for cell survival.

10.1128/mSphere.00475-19.2 FIG S1 Growth curves of wild-type 427 (orange line) and the 3×Ty-TbL11 RNAi (blue line) cell lines. Download FIG S1, TIF file, 0.3 MB. Copyright © 2019 Jaremko et al. 2019 Jaremko et al. This content is distributed under the terms of the Creative Commons Attribution 4.0 International license.

### Loss of TbL11 alters TbL5 and P34 and P37 levels.

**FIG 2:**
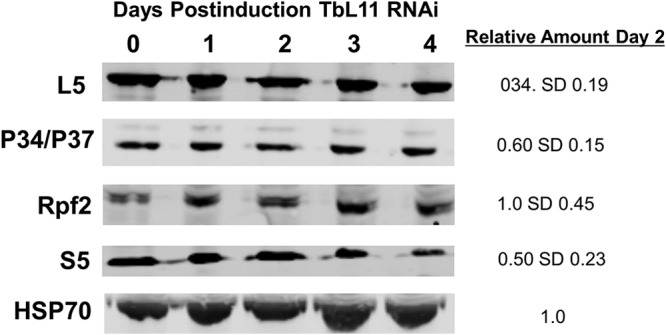
Loss of TbL11 decreases TbL5 and P34 and P37 levels. Western blot analysis was performed using cell extracts collected at days 0 to 4 after induction of TbL11 RNAi. Antibodies were used to probe for each specific protein as indicated beside each blot, with HSP70 used as a loading control. Analyses were performed on three biological replicates, with representative samples shown, and average values with SDs were calculated.

Based on studies from yeast (6) and our previous work with T. brucei (11, 23), we were interested in examining the impact of loss of TbL11 on the other protein members of the 5S RNP. There was a notable, and statistically significant, decrease in protein levels of TbL5 (0.34 [SD, 0.19; *P* = 0.027] at 2 days postinduction relative to uninduced cells) and P34 and P37 (0.60 [SD 0.15; *P* = 0.041]). However, there was no change in levels of TbRpf2 (1.0 [SD, 0.45; *P* = 0.98]), another 5S RNP protein. Furthermore, levels of S5 (0.50 [SD, 0.23; *P* = 0.062), a 40S ribosomal protein, saw a small, but statistically insignificant, decrease (Fig. 2 and Fig. S2). Taken together, these findings show that loss of TbL11 strongly impacts TbL5 and P34 and P37, suggesting an interdependency between TbL11 with TbL5 and P34 and P37.

10.1128/mSphere.00475-19.3 FIG S2 Quantification and graphical representation of changes in protein levels during RNAi induction. (A) Graph showing average values and SDs in levels of 3×Ty-TbL11 upon induction of TbL11 RNAi compared to levels at day 0. (B) Graph showing average values and SDs in 5S RNP proteins upon induction of TbL11 RNAi compared to levels at day 0. Download FIG S2, TIF file, 0.4 MB. Copyright © 2019 Jaremko et al. 2019 Jaremko et al. This content is distributed under the terms of the Creative Commons Attribution 4.0 International license.

### TbL11 knockdown decreases 60S and 80S subunits and generates halfmer peaks.

**FIG 3:**
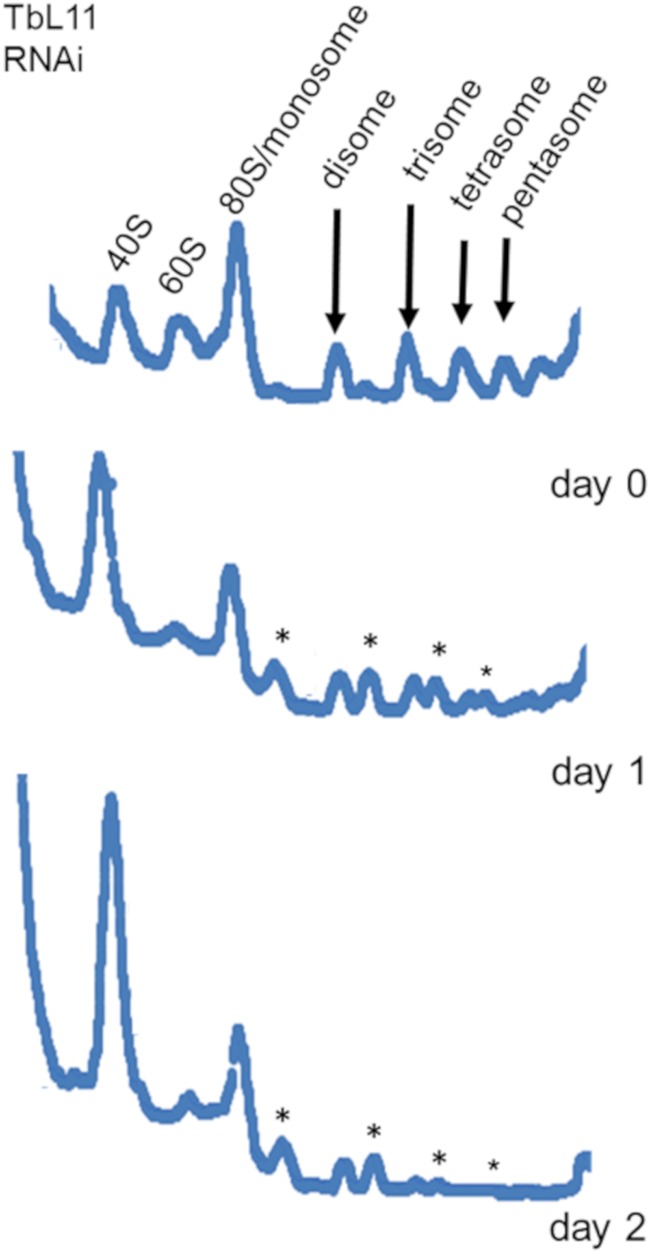
Knockdown of TbL11 decreases 60S and 80S subunits and generates halfmer peaks. Cells were collected at days 0 to 2 after induction of RNA interference knockdown of TbL11, and polysome profile analyses were performed on extracts from these cells. Polysome peaks are indicated by arrows, and halfmer peaks are indicated by asterisks. Analyses were performed on three biological replicates, and representative tracings are shown.

Depletion of ribosomal proteins often leads to reduced levels of assembled ribosomes and polysomes in eukaryotic cells (24). We therefore analyzed the effect that loss of TbL11 (from the untagged cell line) has on the distribution of ribosomal subunits, monosomes (80S particle), and polysomes. After induction of TbL11-specific RNAi, levels of polysomal peaks decreased in comparison to those for uninduced cells (Fig. 3, arrows). The magnitude of this effect became greater as induction progressed and cells became more severely depleted in TbL11. In addition, depletion of TbL11 correlated with the appearance of peaks of intermediate density between particles containing *n* ribosomes and particles containing *n* + 1 ribosomes. These intermediate peaks represent instances of a 40S subunit binding the 5′ end of mRNA, scanning to an initiation codon, but then being unable to recruit a 60S subunit to form an initiation-competent monosome and begin translation. Therefore, “halfmers” can be detected on the sucrose gradient (Fig. 3, asterisks). This is consistent with a destabilizing effect on components of the 60S subunit that disrupts the stoichiometric balance between 60S and 40S subunits. The magnitude of this effect also becomes greater as induction progresses. Finally, we observed an increase in the material absorbing at 260 nm cosedimenting with the 40S subunit. This increase suggests that components of 40S subunits largely remain stable in the absence of TbL11.

### 25/28S and 5.8S rRNA processing is disrupted by loss of TbL11.

**FIG 4:**
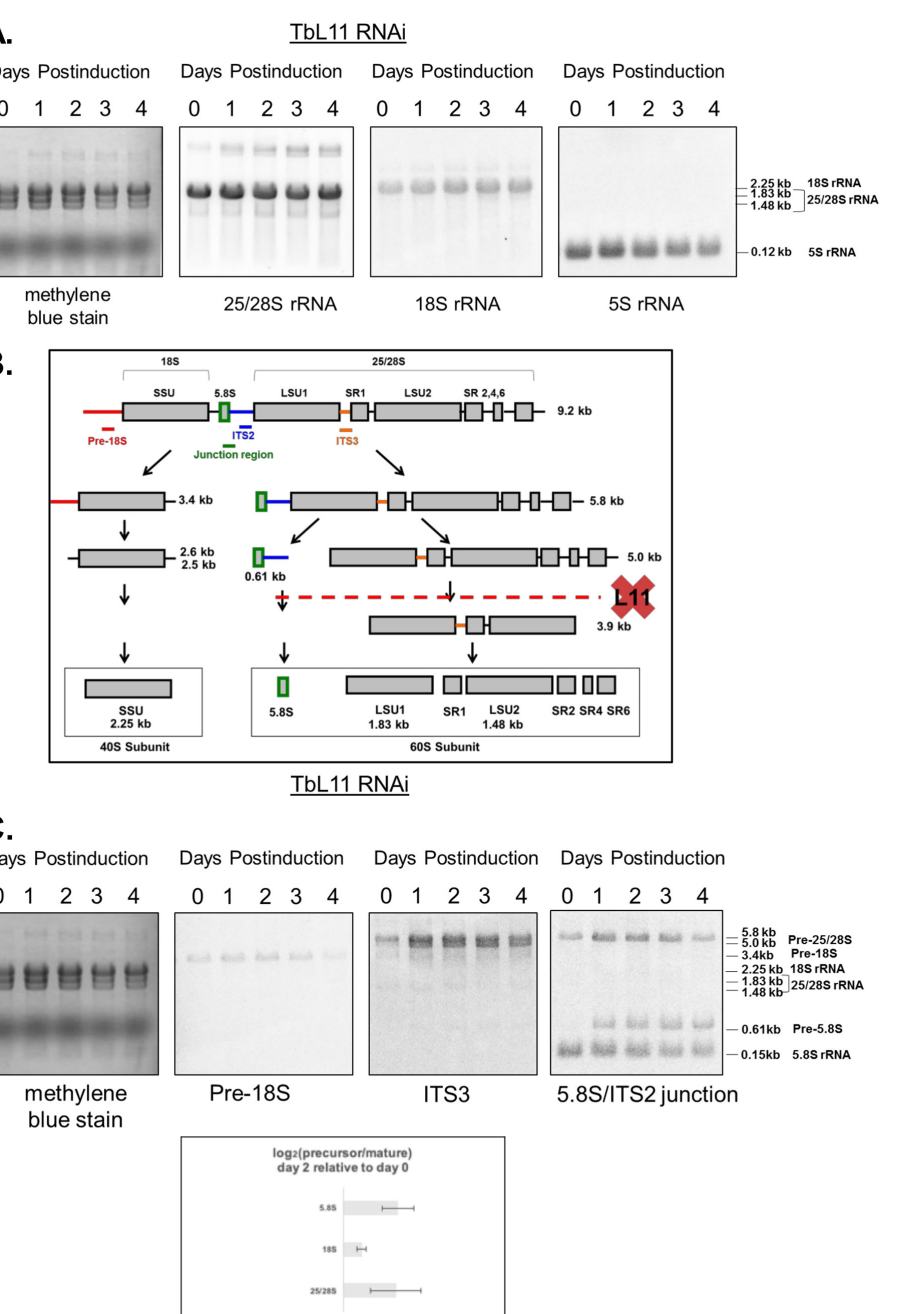
Loss of TbL11 disrupts stages of 25/28S and 5.8S rRNA processing. Following 0 to 4 days of TbL11 RNA interference induction, total RNA was extracted from cells, stained with methylene blue, and then probed for various mature rRNAs (A) or rRNA processing intermediates (C) as labeled. Log_2_ values from ratios of precursor to mature rRNAs on day 0 were substracted from the corresponding log_2_ values on day 2 postinduction (C). Panel B shows a simple schematic of rRNA processing showing the proposed sites of processing interrupted by loss of TbL11 (43). Analyses were performed on three biological replicates, with representative blots shown.

Defects in ribosome biogenesis of the 60S subunit suggest that steady-state levels of rRNAs in the 60S subunits could be affected in TbL11-depleted cells (14). We analyzed total RNA from uninduced and induced cells by Northern blotting using probes directed against mature rRNAs (Fig. 4A). By day 3 postinduction, the steady-state levels of 25/28S rRNA and 5S rRNA experienced a relative decrease (0.8 of day 0, normalized to average fold change), while the signal from 18S rRNA experienced a corresponding 10% increase, a likely result of loading equivalent amounts of total RNA per lane. By day 4, the cells partially recovered from these effects.

Defects in ribosome biogenesis suggest a lack of proper processing of one or more rRNA intermediates. In T. brucei, rRNA processing exhibits unique peculiarities (Fig. 4B), specifically in the arm of the pathway leading to the formation of 25/28S rRNA, which is ultimately processed into six fragments, the largest of which are termed LSU1 and LSU2. We investigated rRNA processing by analyzing specific intermediate species throughout the time course of RNAi for TbL11 (Fig. 4C). A probe directed against pre-18S located upstream of the small subunit (SSU) allowed us to detect the precursor of the 18S rRNA (Fig. 4C, second blot). Probe ITS3 identified 5.0-kb and 5.8-kb intermediates in the 25/28S pathway (Fig. 4C, third blot), and a probe directed against the ITS2/5.8S rRNA junction allowed us to detect a 0.61-kb precursor to 5.8S rRNA. Analysis of the ratio of precursor to mature species for each rRNA (Fig. 4C, bottom) shows that the precursors of the 60S pathway preferentially accumulate when TbL11 is depleted. On day 2 of the induction, the log_2_ ratio of the 5.0-kb precursor to 25/28S mature rRNA was 1.8 (SD, 0.8), and the log_2_ ratio of the 0.61-kb precursor to 5.8S mature rRNA was 1.8 as well (SD, 0.5). However, in the case of the 40S pathway, the log_2_ ratio of pre-18S rRNA to mature 18S rRNA was only 0.6 (SD, 0.2).

### TbL11 is a member of the T. brucei 5S RNP and interacts with trypanosome-specific protein P34 and other 5S RNP members.

**FIG 5:**
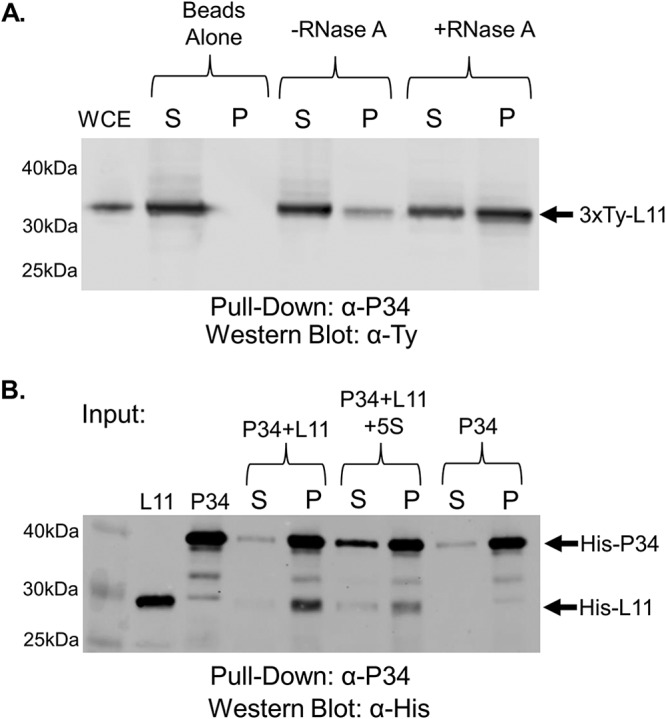
TbL11 interacts *in vivo* and *in vitro* with P34. (A) A whole-cell extract (WCE) was prepared from 3×Ty-TbL11 cells, and coimmunoprecipitations were performed using anti-P34/P37 antibody. The resulting immunoprecipitates were then analyzed via anti-Ty Western blotting. (B) Recombinant TbL11 was incubated with recombinant P34 in an anti-P34/P37 coimmunoprecipitation assay with or without addition of *in vitro*-transcribed 5S rRNA. Western blotting was performed using anti-His antibody. Blots are representative of three biological replicates, and average values with SDs were calculated. S, supernatant; P, pellet. A total of 10 μg of WCE was loaded in the WCE lane.

We next examined whether TbL11 was also a member of the 5S RNP. We performed immunoprecipitations from cell lysates using antibodies specific for P34 and P37, TbL5, or TbRpf2 and probed for the presence of TbL11. We began by incubating a whole-cell extract (WCE) with control beads (lacking antibody) and did not observe a nonspecific interaction between TbL11 and the beads (Fig. 5A, beads alone). We next incubated a whole-cell extract with anti-P34 beads and observed TbL11 weakly coimmunoprecipitating in the pellet fraction with P34 and P37 *in vivo* (Fig. 5A, −RNase A). This interaction was significantly enhanced (0.16 [SD, 0.09] to 0.53 [SD, 0.03; *P* = 0.009] with addition of RNase A) by the addition of RNase A to digest cellular RNA (Fig. 5A, +RNase A).

Using recombinantly expressed P34 and TbL11 (Fig. 5B, L11 and P34 input) in a coimmunoprecipitation assay, we observed that P34 and TbL11 directly interact in the absence of other factors (Fig. 5B, P34 + L11). Furthermore, this interaction seemed to be slightly, though not significantly, reduced (0.96 [SD, 0.02] to 0.85 [SD, 0.11; *P* = 0.16] after addition of 5S rRNA) by the addition of 5S rRNA, supporting our *in vivo* findings (Fig. 5B, P34 + L11 + 5S rRNA). This indicated that 5S rRNA may be partially responsible for inhibiting the P34-TbL11 interaction, but other RNAs must also contribute *in vivo*.

**FIG 6:**
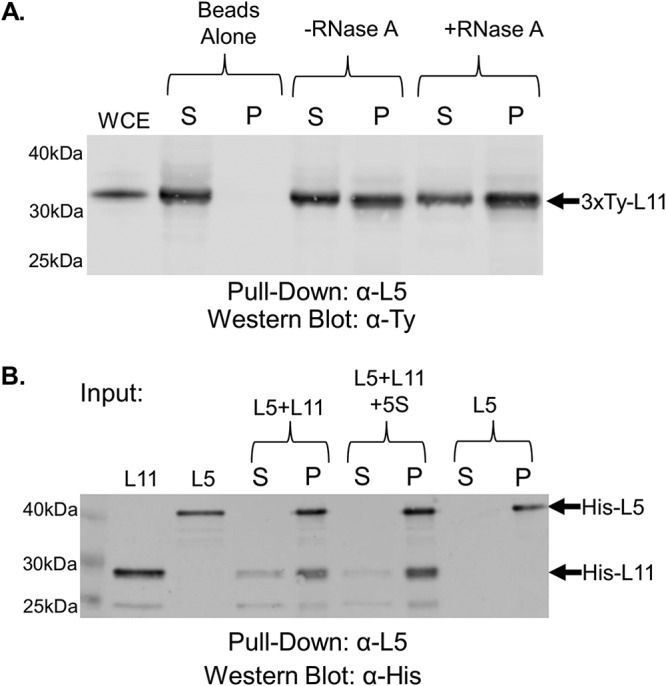
TbL11 interacts *in vivo* and *in vitro* with TbL5. (A) A whole-cell extract was prepared from 3×Ty-TbL11 cells, and coimmunoprecipitations were performed using anti-TbL5 antibody. The resulting immunoprecipitates were then analyzed via anti-Ty Western blotting. (B) Recombinant TbL11 was incubated with recombinant TbL5 in an anti-TbL5 coimmunoprecipitation assay with or without addition of *in vitro*-transcribed 5S rRNA. Western blotting was performed using anti-His antibody. Blots are representative of three biological replicates, and average values with SDs were calculated. A total of 10 μg of WCE was loaded in the WCE lane.

We next examined TbL5, via coimmunoprecipitation using a whole-cell extract, and showed an interaction between TbL11 and TbL5 (Fig. 6A, −RNase A) that was slightly, though not significantly, enhanced (0.53 [SD, 0.1] to 0.67 [SD, 0.18; *P* = 0.11]) by the addition of RNase A (Fig. 6A, +RNase A). We examined the same interaction using *in vitro* coimmunoprecipitation and saw that TbL11 and TbL5 directly interacted (Fig. 6B, L5 + L11) and that this interaction was unchanged (0.92 [SD, 0.11] to 0.90 [SD, 0.14; *P* = 0.85]) by the inclusion of 5S rRNA (Fig. 6B, L5 + L11 + 5S rRNA).

**FIG 7:**
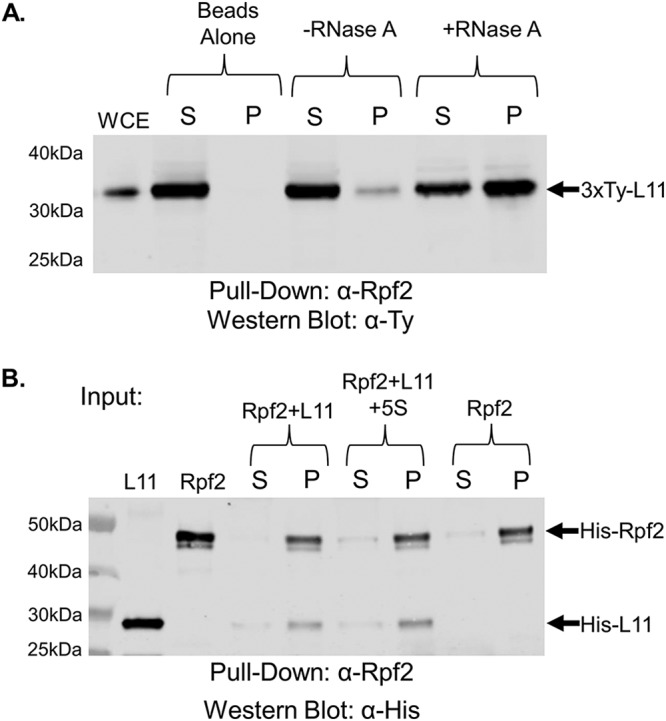
TbL11 interacts *in vivo* and *in vitro* with TbRpf2. (A) A whole-cell extract was prepared from 3×Ty-TbL11 cells, and coimmunoprecipitations were performed using anti-TbRpf2 antibody. The resulting immunoprecipitates were then analyzed via anti-Ty Western blotting. (B) Recombinant TbL11 was incubated with recombinant TbRpf2 in an anti-TbRpf2 coimmunoprecipitation assay with or without addition of *in vitro*-transcribed 5S rRNA. Western blotting was performed using anti-His antibody. Blots are representative of three biological replicates, and average values with SDs were calculated. A total of 10 μg of WCE was loaded in the WCE lane.

Finally, we examined if there was an interaction between TbL11 and TbRpf2. We saw an interaction between the two proteins *in vivo* (Fig. 7A, −RNase A) that was strongly and significantly increased (0.07 [SD, 0.05] to 0.58 [SD, 0.05; *P* = 0.0002]) with the addition of RNase A (Fig. 7A, +RNase A). Next, using recombinantly expressed protein we saw a direct interaction between TbL11 and TbRpf2 (Fig. 7B, Rpf2 + L11) that was not altered by the addition of 5S rRNA (Fig. 7B, Rpf2 + L11 + 5S rRNA; 0.86 [SD, 0.004] to 0.83 [SD, 0.11; *P* = 0.6]). All of these findings confirm that ribosomal protein TbL11 is an additional member of the T. brucei 5S RNP.

### TbL11 is structurally similar to homologues in other eukaryotes but has divergent characteristics.

**FIG 8:**
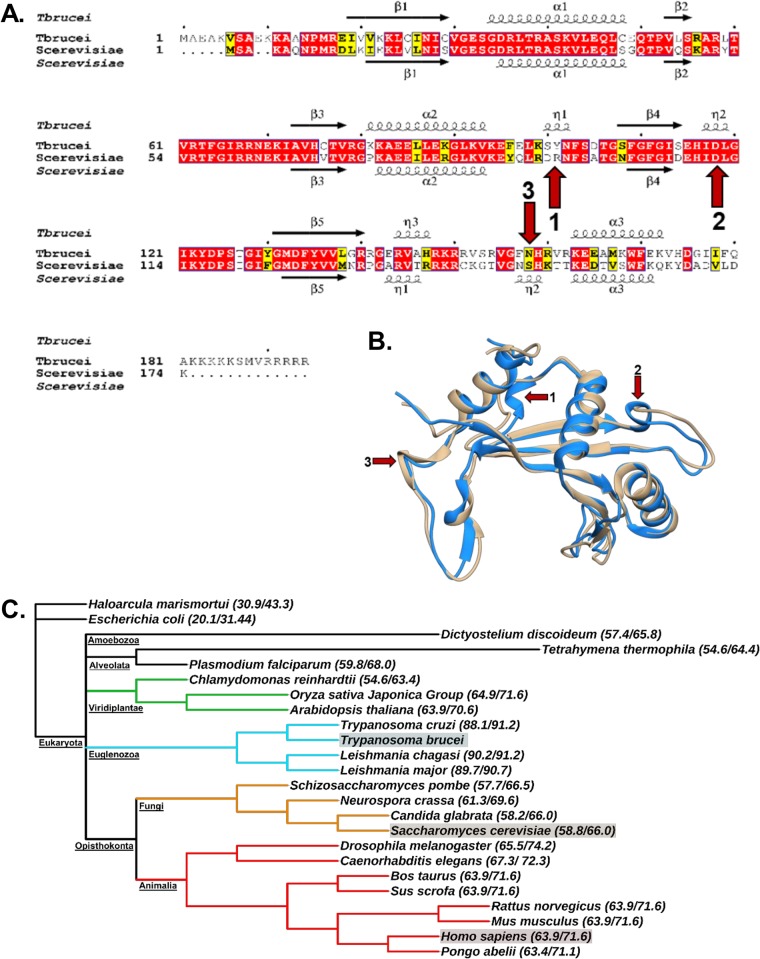
TbL11 is structurally similar to homologues in other eukaryotes but has divergent characteristics. (A) Primary and secondary structural alignment of T. brucei and S. cerevisiae L11 proteins, with arrows highlighting structural differences. (B) Tertiary structures of T. brucei L11 (blue; RCSB PDB code 4V8M) and S. cerevisiae L11 (tan; RCSB PDB code 4V88) with arrows indicating structural differences. (C) Cladogram depicting the levels of evolutionary separation between T. brucei, H. sapiens, and S. cerevisiae, the model yeast. Percent identity and percent similarity of L11 with T. brucei are presented beside each organism name.

We next examined if there was a structural basis for the unique features of the TbL11 relative to other eukaryotic L11 proteins. We began by comparing the primary and secondary structures of TbL11 to those of S. cerevisiae (Fig. 8A). There is a great deal of homology (58% identity and 66% similarity) between T. brucei and S. cerevisiae at the level of primary and secondary structures, which is consistent with the role of L11 as an essential ribosomal protein (25, 26). However, there are also portions of the secondary and tertiary structures that vary between the two proteins (Fig. 8A and B, red arrows), specifically the presence of additional 3_10_-helices, as noted, and greater length and sequence divergence at the N terminus and C terminus of the T. brucei homologue. To further examine differences in L11 across multiple species, the percent identity and percent similarity of TbL11 to homologues in a range of other organisms were calculated (Fig. 8C). From this analysis, it is clear that despite the high homology of L11 across eukaryotes (approximately 60% identity on average), there is an especially tight cluster of identity within the *Kinetoplastidae* (approximately 90% identity [Fig. 8C, blue branches]). These findings support a potential for divergent properties among the *Kinetoplastidae* in an otherwise highly conserved process and highlight the limitations of largely relying on yeast as a model organism.

## DISCUSSION

Eukaryotic ribosome biogenesis is a complex process, and our understanding of this constantly evolving field of study has largely been informed by experiments in S. cerevisiae. While yeast as a model organism provides a strong framework for our knowledge of this complicated process, it is unlikely that ribosome biogenesis is identical in all other eukaryotes. For example, the published structure of the mature T. brucei ribosome, which features many components homologous to those in the yeast ribosome, also includes unique elements such as extended protein and rRNA expansion segments and additional processing of the 25/28S rRNA (27). These differences highlight the need to examine ribosome biogenesis in greater detail in eukaryotes other than S. cerevisiae to provide a better understanding of this process. Toward this end, one important component of ribosome biogenesis that our laboratory has been examining is how the 5S RNP differs in T. brucei and yeast.

Work from our laboratory has identified the trypanosome-specific proteins P34 and P37 as integral members of the T. brucei 5S RNP. These proteins are unique to the *Trypanosomatidae* and essential to the survival of T. brucei, and loss of these proteins disrupts ribosome biogenesis (7). Furthermore, P34 and P37 are important for 5S rRNA abundance (7) and have direct interactions with 5S rRNA (8, 9), as well as the T. brucei homologues of L5 (10), Rpf2 (11), and Rrs1 (12). Further work from our laboratory with TbL5 and TbRpf2 has characterized unique properties that set them apart from their homologues in S. cerevisiae. Specifically, TbL5 directly interacts with P34 and P37 (10) and has differences in residues that are vital for 5S rRNA binding in S. cerevisiae (10), and loss of TbL5 elicits a compensatory increase of P34 and P37 in T. brucei (23). We have also shown that TbRpf2 not only has interactions with trypanosome-specific proteins P34 and P37 but also can be expressed as a stable, functional protein in the absence of TbRrs1, a finding that is in direct contrast to studies with yeast (11). This body of work has laid the framework for the understanding of an integral part of ribosome biogenesis in T. brucei that contains essential, organism-specific characteristics. However, as questions yet remain about the potential role that the other 5S RNP proteins might have in this complex, we set out to characterize the T. brucei homologue of L11.

We began by generating an RNAi cell line and saw that targeted depletion of TbL11 (Fig. 1B) resulted in rapid loss of cell growth (Fig. 1A) and aberrant morphology (Fig. 1C), indicating that TbL11 is essential in T. brucei. Interestingly, loss of TbL11 impacted TbL5 and P34 and P37, which had a notable, and statistically significant, decrease in protein abundance (Fig. 2). This is similar to loss of TbL5, which resulted in a general decrease of 60S ribosomal proteins (23), and loss of TbRpf2, which saw a decrease in levels of TbL5 and P34 and P37 (11). We next examined if TbL11 had a role in ribosome biogenesis. Polysome profiles using TbL11 RNAi cells revealed a defect in 60S subunit maturation (Fig. 3), and analysis of the ratio of precursor to mature RNAs showed a relative accumulation of precursors in the 25/28S rRNA pathway and the 5.8S rRNA pathway, while the ratios for the 18S rRNA pathway were not affected to the same degree (Fig. 4). These findings from both the polysome profiles and rRNA processing indicate that TbL11 has a critical role in 60S subunit maturation. These data are consistent with previously published observations of the role of L11 in S. cerevisiae (13) and Homo sapiens (28) ribosome biogenesis. However, that itself is somewhat surprising given the unique aspects of ribosome biogenesis in T. brucei, especially in the context of the 5S RNP as previously shown by work from our laboratory. This, then, suggests that TbL11 might have some parasite-specific characteristics that allow it to maintain a similar functionality in T. brucei despite the trypanosome-specific aspects of ribosome biogenesis.

We therefore set out to use more direct methods to determine if TbL11 was part of the 5S RNP in T. brucei and if it shared any unique interactions with other 5S RNP members. We identified TbL11 as a 5S rRNA binding partner using an affinity capture study, suggesting that it was potentially a member of the 5S RNP. This finding was strengthened by evidence from our previously published data using tagged P34, P37, and TbL5, which consistently pulled down TbL11 (11).

Using coimmunoprecipitation, we showed that TbL11 interacts with P34 and P37 *in vivo* and that this interaction was greatly enhanced by the removal of cellular RNA (Fig. 5A). However, we observed that *in vitro*, while TbL11 and P34 directly interacted, the addition of 5S rRNA had only a minor impact on this interaction (Fig. 5B), suggesting that other RNAs, such as portions of the 25/28S rRNA, might be also responsible for inhibiting this interaction *in vivo*. We next showed that TbL11 and TbL5 interact both *in vivo* (Fig. 6A) and *in vitro* (Fig. 6B). However, unlike for the TbL11-P34 interaction, we saw that the presence of RNA did not have an impact on the TbL11-TbL5 association in either case. Finally, in examining the TbL11-TbRpf2 interaction, we saw that while *in vivo* the presence of RNA greatly inhibited the interaction (Fig. 7A), the addition of 5S rRNA *in vitro* (Fig. 7B) did not impact the direct protein-protein interaction.

In the context of ribosome biogenesis, these varied interactions could be connected to the major conformational shift that occurs in the 5S rRNA as part of the nucleolar ribosome-associating 5S RNP. Studies with yeast have shown that during early stages of assembly, the 5S rRNA is rotated nearly 180° relative to its mature conformation. For maturation to continue past this point of assembly, the 5S rRNA must rotate toward its mature conformation, at which point Rpf2 and Rrs1 must disassociate from the ribosome, allowing for 5S rRNA to complete the rotation into its mature position (29).

With this in mind, TbL11 may initially form interactions with TbL5, P34 and P37, and TbRpf2 prior to the conformational shift of the 5S rRNA. At this early stage, all components of the 5S RNP may interact to help establish the RNA-protein neighborhood that is necessary for the next steps of maturation. However, as the 5S rRNA rotates, it might then expose portions of 25/28S rRNA or induce a conformational shift that leaves this RNA free to bind to TbL11. This binding of 25/28S rRNA could disrupt the TbL11-TbRpf2 and the TbL11-P34 and -P37 interactions. The interruption of the TbL11-TbRpf2 interaction could be necessary to allow removal of TbRpf2-TbRrs1 (transiently associating protein factors), which is required for further 60S maturation. Additionally, the disruption of the TbL11-P34 and -P37 interaction may be required to allow interaction with export proteins such as Xpo1 and Nmd3 (30), which is necessary for transport of the 60S subunit to the cytoplasm. The proposed disruption by 25/28S rRNA is consist with observations that S. cerevisiae L11 binds to helix 84 of the 25/28S rRNA in the mature (31) and assembling (32) ribosomes, with helix 84 initially in a distorted conformation prior to proper 5S rRNA insertion. Thus, the interactions with TbRpf2 and P34 and P37 could be important to help keep TbL11 bound to the nucleolar ribosome-associating 5S RNP until it is able to bind to portions of 25/28S rRNA in their final conformation and reach its final mature configuration as a member of the ribosome-associated 5S RNP.

Another possible explanation of these findings might be similar to observations in H. sapiens that the 5S RNP plays a role in helping resolve the disruption of proper ribosome biogenesis through interactions with p53 (33). Therefore, it is possible that the enhanced protein-protein interactions that occur upon degradation of RNA in the T. brucei 5S RNP might be part of a response to interruption of ribosome biogenesis. As the partially assembled 60S subunit degrades, the resulting loss of rRNA could push the 5S RNP members to aggregate as part of a “disruption-associated” 5S RNP complex. Rather than having a role in physiologic assembly of the 60S subunit, this disruption-associated 5S RNP could play a role in downstream signaling to resolve interrupted ribosome biogenesis and restore a homeostatic state. Furthermore, this disruption-associated 5S RNP complex, along with the other explanation presented above, could be one of several roles that the 5S RNP plays under different conditions within the cell. This offers two possible explanations to the observation that some RNA species have a dramatic impact on protein-protein interactions that occur within the T. brucei 5S RNP.

Finally, we compared the structures of the T. brucei homologue of L11 and S. cerevisiae L11. An alignment of the primary and secondary structural features of the two proteins (Fig. 8A) shows a relatively high degree of homology between both the sequences and the secondary structures of the two proteins. However, the alignment also highlights some differences, such as the presence of extended and relatively dissimilar N and C termini of the T. brucei protein, as well as regions of differing inclusion of 3_10_-helices in the secondary structures between the two organisms (Fig. 8A, red arrows). These deviations are of interest, as the termini of T. brucei L11 are situated relatively close to the 5S rRNA in the mature, translation-competent ribosome, a positioning that could allow for interactions with other 5S RNP proteins (27). Furthermore, the inclusion of additional 3_10_-helices (also present in tertiary structures [Fig. 8B]) in L11, change the structural constraints of the protein, which might have a role in forming or breaking interactions during ribosome biogenesis. These 3_10_-helices are of particular interest as they sit near portions of the 25/28S rRNA and the 60S-40S interface, suggesting possible roles in maturation and intersubunit communication (27). To better understand this comparison in a larger context, we aligned L11 primary sequences from a range of eukaryotes to determine how much these proteins differed from TbL11 **(**Fig. 8C). Interestingly, we observed that while ribosomal protein L11 has relatively high conservation across eukaryotes, there is an especially high percent identity within the *Kinetoplastidae*. This could support the presence of trypanosome-specific characteristics of TbL11 that we have observed in this study and suggests that these might be important to the unique aspects of ribosome biogenesis in the *Kinetoplastidae* that might be used to develop future pan-trypanocidal treatments.

Our laboratory has focused on examining one critical aspect of ribosome biogenesis, the formation and incorporation of the 5S RNP, and how it differs in the human pathogen T. brucei compared to the model organism S. cerevisiae. In characterizing TbL11 for the first time, we identified several of its noteworthy properties in this parasite. We showed that it directly interacts with TbL5 and TbRpf2, reinforcing findings in model organisms. However, it is interesting that these interactions still occur despite the parasite-specific characteristics of these proteins (10, 11, 23) and of T. brucei ribosome biogenesis as a whole. In addition, we have shown a unique characteristic of TbL11 through evidence that it directly interacts with the trypanosome-specific proteins P34 and P37. The addition of TbL11 as an interacting partner with these parasite-specific proteins helps solidify their role as unique members of this critical checkpoint of ribosome biogenesis and strongly reinforces how this process can vary between organisms. Furthermore, the increasing focus on the involvement of the 5S RNP in human pathologies such as Huntington disease (34), various ribosomopathies (20, 28, 35), and numerous types of cancer (21, 33, 36) makes it likely that 5S RNP-targeted therapeutics will soon be under development. By expanding our understanding of ribosome biogenesis to incorporate the diversity of characteristics that can be observed in organisms such as T. brucei, we can begin to better understand how to integrate findings from various related eukaryotic systems to target human African trypanosomiasis and other diverse human diseases.

## MATERIALS AND METHODS

### *In vitro* transcription of 5S rRNA.

5S ribosomal DNA (rDNA) was PCR amplified and *in vitro* transcribed as previously published (9). DNase I (Life Technologies) was added to remove template DNA, and NucAway spin columns (Ambion) were used to remove unincorporated nucleotides and any proteins.

### 5S rRNA affinity capture assays.

T. brucei 5S rRNA was transcribed as described above, and following incubation with DNase I (Life Technologies) and ethanol precipitation, 50 pmol of the product was 3′ end labeled with desthiobiotinylated cytidine bis-phosphate (Life Technologies) following the manufacturer’s directions. A total of 50 μl/reaction of streptavidin-bound magnetic beads (Life Technologies) was incubated with 50 pmol of biotinylated 5S rRNA, and then 150 μg of T. brucei 427 procyclic whole-cell extract (11) was incubated with the beads at 4°C for 60 min. Beads were washed, resuspended in 25 μl of SDS sample buffer, and electrophoresed on SDS bis-Tris acrylamide gels. The gels were stained with SyproRuby (Thermo), and the protein bands were excised and identified using mass spectrometry (Thermo Scientific Orbitrap Fusion; Fred Hutchinson Cancer Research Center, Seattle, WA). The sorting and analysis were performed using Proteome Discoverer 2.2 (Thermo Fisher Scientific) and Microsoft Excel.

### Generation of RNAi and Ty-tagged cell lines.

The full TbL11 gene product (TriTryp accession numbers Tb927.9.7590 and Tb927.9.7620) was PCR amplified (Table 1) from genomic DNA, doubly digested, and ligated into the p2T7-177 expression plasmid (37). The resulting plasmid was linearized and transfected (Amaxa Nucleofactor II) into the procyclic 29-13 strain (38, 39), and RNAi cells were selected with phleomycin (2.5 μg/ml). Growth curves were calculated in the presence or absence of tetracycline (2.5 μg/ml) and are based on three biological replicates, with average values and standard deviations shown.

**TABLE 1:** Sequences of primers used in this study

Primer/purpose	Sequence
L11 RNAi For(BamHI)/RNAi construct	5′-CAC CAC AGC CAG GAT CCG ATG GCT GAG GCA AAG GTG
L11 RNAi Rev(HindIII)/RNAi construct	5′-TAT GCG GCC GCA AGC TTT TAA CGG CGG CGA CG
3×Ty-L11 For	5′-TGAACTTTACCGTAATGCACTCCCTCCACTGTCCT GCGCATGTAGTGGCTGTAGAAAGGGATAGCCTCC GTTCTTGACAAGTATAATGCAGACCTGCTGC
3×Ty-L11 Rev	5′-TTAATGCAGAGCTTTTTTACGACAATCTCCCGCAT CGGGTTTGCCGCCTTCTTCTCAGCGCTCACCTTT GCCTCAGCCATATCCAAGGGATCTTGATT
L11 For/pTrcHis construct	5′-ATG GCT GAG GCA AAG GTG A
L11 Rev/pTrcHis construct	5′-TTA ACG GCG GCG ACG G
T3-5S For/*in vitro* transcription	5′-ATT AAC CCT CAC TAA AGG GTA CGA CCA TAC TTG GCC
5S Rev/*in vitro* transcription	5′-AGA GTA CAA CAC CCC GGG T

Wild-type 427 cells or the TbL11 RNAi cell line was used for preparation of 3×Ty-TbL11-tagged cell lines as previously described (Table 1; plasmids generously provided by Sam Dean) (40). Cells were grown under selection using puromycin (1 μg/ml), and extreme limiting dilution was used to prepare clonal lines. Tagged cell lines were used to determine the degree of knockdown of TbL11 and *in vivo* coimmunoprecipitation since we were unable to generate a specific antibody for TbL11. Growth of tagged cell lines and that of untagged cell lines were equivalent, as were levels of expression of 5S RNP and S5 proteins.

### Western blotting.

A whole-cell lysate was prepared as previously described (11). Fifteen micrograms of extract was transferred to a 0.4-μm nitrocellulose membrane (Bio-Rad) and proteins were analyzed using antibodies for TbL5 (10) and P34 and P37 (41) at dilutions of 1:1,000, anti-Ty (Thermo Fisher) at a dilution of 1:2,000, HSP70 antibody (42) at a dilution of 1:20,000, and TbRpf2 (11) and S5 (Abnova) antibodies at a dilution of 1:500 in Odyssey blocking buffer (Li-Cor Technologies). For *in vitro* coimmunoprecipitations, anti-His (Thermo Fisher) was used at a dilution of 1:1,000 in Odyssey blocking buffer (Li-Cor Technologies). Li-Cor secondary antibodies were used to allow quantification of the signals in Image Studio software (Li-Cor Technologies); the signals are directly proportional to the amount of target protein. All data shown are representative blots from three biological replicates and data were calculated relative to HSP70, were compared to those for uninduced cells, and are presented as averages with standard deviations. Statistical significance was calculated for the Western blots during RNAi induction by a paired two-tail *t* test on each protein comparing levels at day 2 postinduction and day 0 using Excel (Microsoft).

### Differential interference contrast microscopy.

Uninduced and induced TbL11 RNAi cells were prepared for DIC microscopy by fixation, mounting using Prolong gold antifade reagent with 4′,6-diamidino-2-phenylindole (DAPI; Life Technologies), and imaging with a Zeiss Axioimager M2 microscope and Volocity 6.1 Acquisition software. Experiments were performed in triplicate, with representative results shown.

### Polysome profiles.

Isolation of polysomes, monosomes, and ribosomal subunits was performed by ultracentrifugation of 5 × 10^8^ cells on 10 to 40% sucrose gradients as previously described (7). Equivalent *A*_260_ units were loaded for each sample. Profiles were collected at the days indicated, with a representative sample from three biological replicates shown.

### Total RNA extraction and mRNA Northern blotting.

TRIzol was used to extract cellular RNA by following the manufacturer’s instructions, and 5 μg of that RNA was used for Northern analysis using specific probes against rRNA intermediates (23) as previously published (43). Images were taken using a Typhoon phosphorimager (GE Technologies) and quantified with ImageJ software (44). Ratio analysis of precursors was performed following previously published methodologies (45). All data presented are representative of three biological replicates.

### Cloning and expression of recombinant proteins.

T. brucei whole genomic DNA was used to PCR amplify TbL11 (Tb927.9.7590 and Tb927.9.7620) (Table 1), which was then cloned into the pTrcHis-TOPO-TA vector (Life Technologies) and expressed in Escherichia coli Top10 One Shot cells (Life Technologies). Expression of P34 (46), TbRpf2 (11), and TbL5 (47) was performed using previously generated plasmids. Purification was performed under previously published conditions (10), and proteins were detected via Western blotting using anti-His.

### Coimmunoprecipitations.

Coimmunoprecipitation was performed with either a whole-cell lysate from Ty-tagged L11 cells or recombinant proteins as previously published (47). Since we have previously shown that P34 and P37 are functionally identical (9, 10), P34 was used *in vitro* as representative of both. Bound protein was eluted and unbound protein was ethanol precipitated from the supernatant, followed by resuspension in 1× SDS sample buffer. The entire eluted pellet fraction and the ethanol-precipitated supernatant were electrophoresed and blotted as described above. All data are presented as representative blots from three biological replicates, and data were quantified using Image Studio (Li-Cor Technologies) as the amount in the pellet relative to the sum of the protein in both pellet and supernatant fractions and presented as calculated means and standard deviations. Statistical significance was calculated via two-tailed *t* tests for each of the assays using Excel (Microsoft).

### Structural analyses.

Sequences and structures were obtained from UniProt (Fig. S3) when possible, though some structural information was either extracted from RCSB PDB files or predicted via iTasser software (48). Alignments were performed with Clustal Omega (49), and identity and similarity were calculated using online server software tools available from SIAS (http://imed.med.ucm.es/Tools/sias.html) and SMS (http://www.bioinformatics.org/sms2/ident_sim.html). Taxonomic trees were designed using NCBI Common Tree and modeled using NCBI Tree Viewer and Interactive Tree of Life (50). Alignments of sequences and structures were generated using ESpript (51), and all tertiary information was modeled using Chimera (52).

10.1128/mSphere.00475-19.4 FIG S3 UniProt identifiers or TriTryp accession numbers for comparative structural analyses. Download FIG S3, TIF file, 0.5 MB. Copyright © 2019 Jaremko et al. 2019 Jaremko et al. This content is distributed under the terms of the Creative Commons Attribution 4.0 International license.
