# Supplementary figures and images for: Trypanosoma brucei L11 Is Essential to Ribosome Biogenesis and Interacts with the Kinetoplastid-Specific Proteins P34 and P37
Source: mSphere. 2019 Aug 21;4(4):e00475-19. doi: 10.1128/mSphere.00475-19 (PMC6706469; doi:10.1128/mSphere.00475-19)

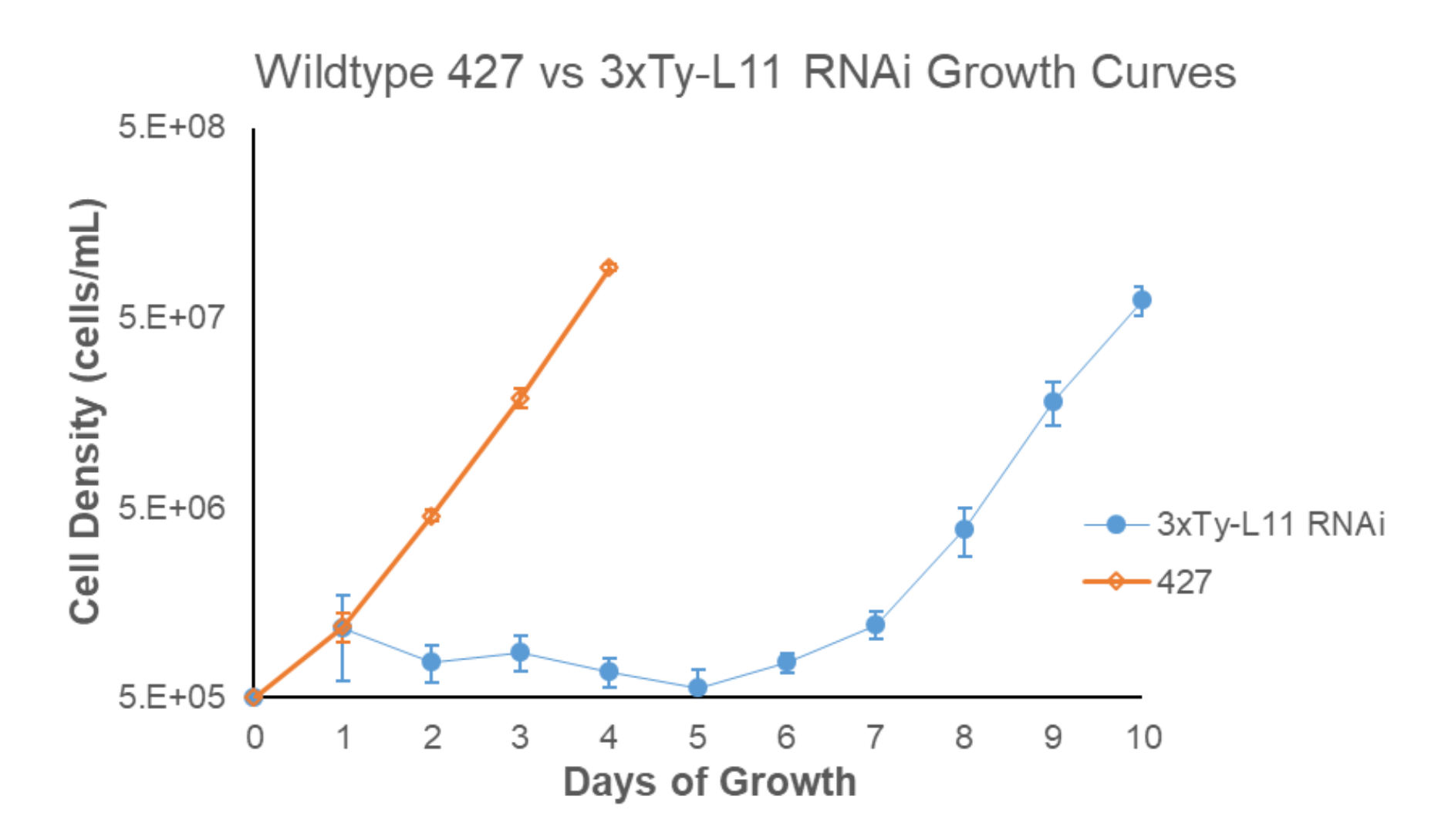

Supplement: FIG S1 [file mSphere.00475-19-sf001.tif]

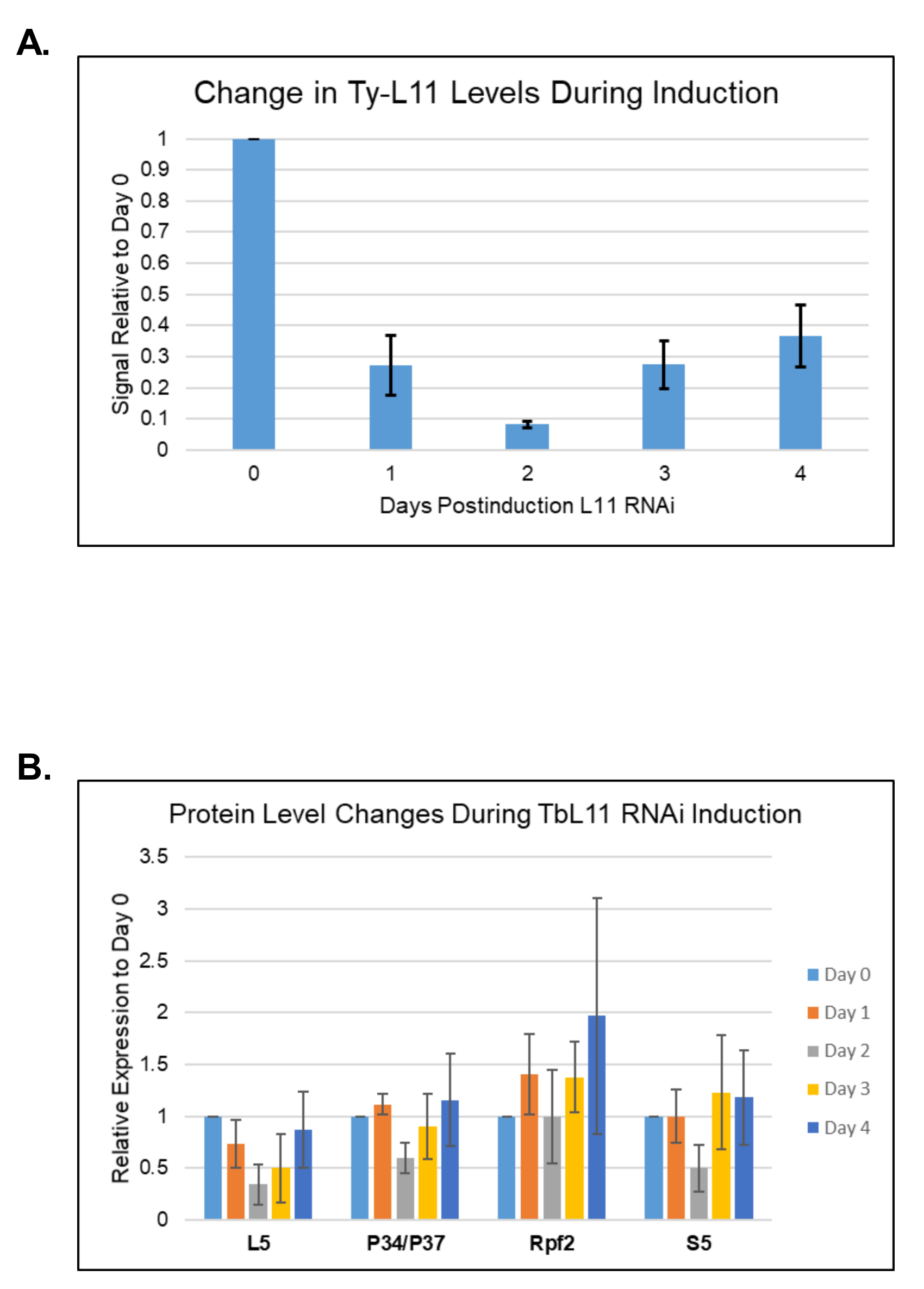

Supplement: FIG S2 [file mSphere.00475-19-sf002.tif]

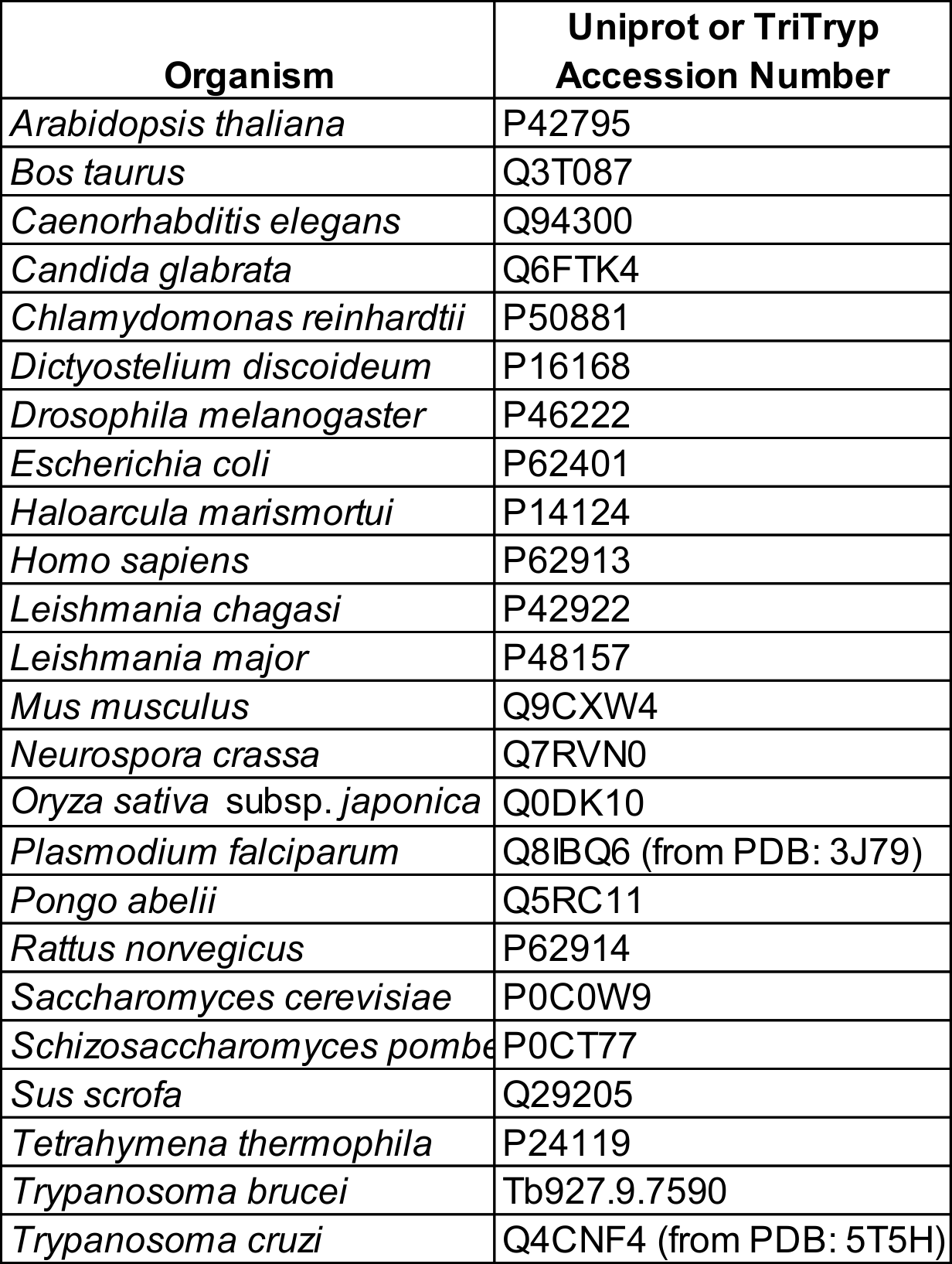

Supplement: FIG S3 [file mSphere.00475-19-sf003.tif]
